# „Kriseninternes Lernen“ und „krisenübergreifendes Lernen“ in der deutschen Kommunalverwaltung: Eine empirische Analyse von Verwaltungshandeln während der Flüchtlingskrise und der Corona-Pandemie

**DOI:** 10.1007/s41358-022-00323-5

**Published:** 2022-06-15

**Authors:** Michael W. Bauer, Jana Otto, Rahel M. Schomaker

**Affiliations:** 1 School of Transnational Governance, European University Institute, Via Cavour 65, 50121 Florenz, Italien; 2 Deutsche Universität für Verwaltungswissenschaften Speyer, Freiherr-vom-Stein-Str. 2, 67324 Speyer, Deutschland; 3 CUAS Villach, Europastraße 4, 9524 Villach, Österreich; 4 Deutsches Forschungsinstitut für öffentliche Verwaltung Speyer, Freiherr-vom-Stein-Str. 2, 67324 Speyer, Deutschland

**Keywords:** Verwaltungshandeln, Krise, Lernen, COVID-19, Fluchtmigration, Resilienz, Administrative action, Crisis, Learning, COVID-19, Refugee migration, Resilience

## Abstract

Krisen testen die Leistungsfähigkeit von Verwaltungen unter Realbedingungen. Vor diesem Hintergrund analysiert der vorliegende Beitrag Reaktion der deutschen Kommunalverwaltung auf die Fluchtmigration zwischen 2015 und 2017 und auf die erste Welle der COVID-19-Pandemie in 2020. Mit Blick auf die Debatte zum organisationalen Lernen in Ausnahmesituationen liegt der Schwerpunkt der Analyse auf der Rolle administrativer Netzwerke sowie der Lernfähigkeit von öffentlichen Behörden während sowie zwischen Krisensituationen. Die Auswertung zweier Umfragen unter Mitarbeitern der deutschen Kommunalverwaltung zeigt erstens, dass die Qualität der verwaltungsinternen und der zivilgesellschaftlichen Vernetzung von zentraler Bedeutung für administrative Krisenperformanz sind. Zweitens korrespondiert Leistungsfähigkeit in Krisen mit der Bereitschaft sowie mit der Fähigkeit, Lehren aus früheren Krisen zu ziehen.

## Abstract (en)

Crises constitute a test of the efficiency of the administrations under real conditions. This is where this article departs, comparatively analyzing the recent “migration crisis” and first wave of the COVID pandemic. Against the backdrop of the debate on organizational learning of public administration in exceptional situations, the analysis focuses on the role of administrative networks and the ability to learn during and between crises. The evaluation of two surveys among employees of German local government shows firstly that the quality of networking within the administration and civil society is of central importance for administrative crisis performance. Second, successful crisis performance corresponds to the willingness and organizational ability to draw lessons from previous crises.

## Einführung

Die Dramatik der gegenwärtigen Corona-Situation in Kombination mit der Aufnahme hunderttausender Flüchtlinge aus der Ukraine lassen deuten an, dass staatliche Verwaltungen regelmäßig mit Herausforderungen mit Krisencharakter konfrontiert sind. Ereignisse wie der Fallout im Zuge der Reaktorkatastrophe von Tschernobyl, der Zustrom von Bürgern aus der DDR 1989 und die Aufnahme von Bürgerkriegsflüchtlingen aus dem ehemaligen Jugoslawien Anfang der 1990er-Jahren hatten alle spezifische Charakteristika von außergewöhnlichen und umfassenden Herausforderungen für die deutsche Verwaltung (Czada 2001; Drexler und Czada 1987; Hustedt 2019). Die Frage ist daher, wie Verwaltungen mit diesen Ausnahmesituationen umgehen. Bleiben Krisenerfahrungen singuläre Ereignisse oder können aus ihrer Bewältigung nachhaltig Lehren für die Zukunft gezogen werden? In der Verwaltungswissenschaft wird vor diesem Hintergrund das Konzept des organisationalen Lernens diskutiert (Boin und Lodge 2016; Lalonde 2007; Moynihan 2008, 2009). Erkenntnisse aus zwei jüngeren Krisensituationen, der gestiegenen Fluchtmigration zwischen 2015 und 2017 sowie der Umgang mit der ersten Welle der Corona-Pandemie im Frühjahr 2020, ermöglichen im Folgenden einen auf deutsche Kommunalverwaltungen bezogenen empirischen Vergleich gängiger Annahmen dieser Debatte.

Auch wenn die COVID-19-Pandemie und die sogenannte „Flüchtlingskrise“ andersgeartete Anforderungen an unterschiedliche Teile der Verwaltung stellen beziehungsweise gestellt haben, so sind die behördeninternen Lernprozesse, so unsere Annahme, durchaus vergleichbar. Diese Annahme dient als Ausgangspunkt, um nachfolgend ein empirisches Modell des Krisenlernens zu entwickeln. Dabei sollen organisationale Lerneffekte und das Anpassungsvermögen innerhalb und zwischen Krisen im Mittelpunkt stehen (Moynihan 2008, 2009). Mithilfe von Daten aus zwei Verwaltungssurveys (zur Fluchtmigration der Jahre 2015 bis 2017 und über die Anfangsphase der COVID-19-Pandemie von April bis Mai 2020) werden theoretisch abgeleitete Erwartungen empirisch getestet, um so der Krisenperformanz deutscher Kommunalverwaltungen auf diesen Feldern nachzuspüren. Die empirischen Ergebnisse der vorliegenden politikwissenschaftlichen Verwaltungsstudie legen nahe, dass insbesondere die *Qualität der Vernetzung* einer Verwaltung sowie die *Fähigkeit, aus in früheren Krisen gemachten Erfahrungen zu lernen*, den Behörden in Krisensituationen maßgeblich helfen, ungewöhnliche Herausforderungen „im Ausnahmezustand“ zu meistern.

## Verwaltungshandeln in Krisen: Netzwerke und Interaktives Lernen

Als Krisen werden Ereignisse mit geringem Wahrscheinlichkeitseintritt und großen Auswirkungen für die Gesellschaft bezeichnet (Rosenthal et al. 1989). Sie stellen den Zusammenhalt sowie die Institutionen einer Gesellschaft auf die Probe (Carmeli und Schaubroeck 2008) und gelten als Beispiele für „wicked problems“ vor denen Regierung und Verwaltung immer öfter stehen (Kettl 2006, S. 275). Sie sind gekennzeichnet durch „erhebliche Konsequenzen, begrenzte Zeithorizonte, hohe politische Brisanz, Unsicherheit und Ambivalenz“ (Moynihan 2008, S. 351, Übersetzung der Autoren).

Ein großer Teil der Studien über die Leistungsfähigkeit öffentlicher Verwaltungen in Krisenzeiten kommt zu dem Schluss, dass Bündelung und stärkere Zentralisierung im Hinblick auf Schnelligkeit und Kohärenz von Entscheidungen erstens nützlich und zweitens ohnehin erforderlich sind, weil in Krisenzeiten die Bedeutung starker exekutiver Führung steigt (Boin et al. 2017; Boin und t’Hart 2003; t’Hart et al. 1993; Drennan et al. 2015). Somit würde größere Leistungsfähigkeit in der Krise eher einen hierarchischen Ansatz der Organisations- und Interaktionsstruktur notwendig machen (Moynihan 2009).

Allerdings wird einem hierarchischen Organisationsmodell auch eine größere Anfälligkeit für opportunistische Verhaltensmuster nachgesagt (t’Hart et al. 1993). Wenn möglichst große Einheiten und umfassende Integration von dezentralen Teileinheiten in (bestehende) Hierarchien als notwendig angesehen werden, um eine Krise zu meistern, dann kann das wiederum informelle Grabenkämpfe um Budget und Personal zwischen Unterorganisationseinheiten befördern. Krisenreaktionsfähigkeit würde dann zu einem Nullsummenspiel. Nicht Kooperation, sondern vielmehr die Stärkung der einzelner Organisationseinheiten wäre das Leitbild für eine erfolgversprechende administrative Krisenstrategie (Peters et al. 2011).

Eine Gegenposition nehmen Koordinations- und Kommunikationsmodelle ein (Moynihan 2009). Erfolge in der Krisenbewältigung werden demnach nicht durch Subordination und Zentralisierung gewährleistet, sondern durch Kooperation, Koordination und dem Experimentierpotenzial einer Vielzahl dezentraler Einheiten (Schuppert 2000). An die Stelle von Unterordnung und top-down Steuerung treten Ansätze zur Problemlösung durch vermehrte Interaktion und durch Kommunikation mit relevanten intra-organisationalen Akteuren sowie mit gesellschaftlichen Gruppen der Verwaltungsumwelt. Die Etablierung einer temporären hierarchischen Struktur, um verschiedene Einheiten enger zu koordinieren, steht dabei nicht unbedingt im Widerspruch zu Netzwerkansätzen als solchen, da die Pluralität mehrerer zentralisierter Netzwerke ebenfalls als eine besondere Form von Netzwerk-Governance verstanden werden kann (Moynihan 2009). Netzwerke, die auf dem Prinzip basieren, komplexe Sachverhalte durch dezentrale Strukturen zu managen, um sich effektiver an lokale Gegebenheiten anzupassen, werden in der Regel sogar als äquivalent und häufig als in ihrer Performanz und Resilienz als überlegen angesehen (Duit 2016). Denn der große Vorteil der Einbindung relativ autonomer Akteure zeigt sich nicht zuletzt in einer höheren Implementationseffizienz. Diese kompensiere, so die Argumentation, die im Vergleich mitunter langwierigen Abstimmungsprozesse um ein Vielfaches. Zudem generiert dieses Verfahren eine größere Legitimität und höhere „Ownership“ der gewählten Strategien unter allen Beteiligten. Das ist besonders dann von Bedeutung, wenn die vorgeschlagenen Reaktionen Verantwortlichkeitsebenen und -domänen überschreiten beziehungsweise Strukturveränderungen oder andere Muster der Ressourcenreallokationen notwendig machen (Peters et al. 2011).

Ähnliche Argumente können für eine größere Dezentralisierung und Einbeziehung der Zivilgesellschaft bei der Bewältigung von Krisensituationen ins Feld geführt werden. Komplexität lässt sich nicht beliebig reduzieren – ein Umstand, der mit Blick auf hierarchische Lösungen zu der These führt, dass dezentrale Kooperation unter Beteiligung der Zivilgesellschaft gerade bei „wicked problems“ zielführender sei (Edlefsen und Staemmler 2018; Kettl 2006). Konkret können demnach netzwerkartige Beziehungen, die Behörden, Berufsorganisationen, Verbände und Ehrenamtliche einschließen, dazu beitragen, einer Überforderung der Verwaltung als Organisationshierarchie entgegenzuwirken (Alford 2011; Bovaird 2007; Granovetter 1985). Durch erleichterte Kommunikation im Netzwerk können die Akteure Wissen, Standards und Ressourcen unproblematisch austauschen und so neue Denkweisen, Ansichten und gegebenenfalls gemeinsame Strategien schaffen, wie dies auch durch Co-Design- und Co-Produktionsansätze immer wieder betont wird (Ostrom 1996; Torfing 2016; Probst et al. 2012).

Folgt man diesem Netzwerk-Paradigma, so kann die administrative Krisenreaktion durch Kooperation vielfältig verbessert werden. Innovationen würden wahrscheinlicher und Lösungen, die auf diese Weise in Zusammenarbeit zwischen Gesellschaft und Verwaltung erarbeitet werden, sollten darüber hinaus auf eine breite Akzeptanz von Verwaltung, Zivilgesellschaft und den entsprechenden Programmadressaten treffen (Sack 2016). Netzwerke wären in der Krisenreaktion hierarchischen Organisationsstrukturen überlegen, weil sie größeres Lernpotenzial entwickeln, schneller auf Umweltveränderungen reagieren und Neuerungen durch breitere Akzeptanz leichter ins Werk setzen können (Moynihan 2008; Torfing 2016; Milward und Provan 2006).

Innovation und Lernen sind demnach also eng miteinander verbundene Konzepte. Greifen Organisationen auf Erfahrungen aus der Vergangenheit zurück und lernen daraus, dann könnten, so die Annahme, diese Erfahrungen produktiv auf neue Situationen angewandt werden. Eine in diesem Sinne innovative Verwaltung lenkt die Aufmerksamkeit auf die organisatorische Fähigkeit zur Selbstreflexion und auf intraorganisatorische Lernprozesse (Wollmann 2017; Lewis et al. 2018). Ansätze dieser Provenienz betonen insbesondere die Bedeutung einer systematischen Dokumentationsfunktion über vergangenes Krisenhandeln, stellt doch der Zugriff auf Wissen über fehlgeschlagene oder erfolgreiche Maßnahmen sowie über die Wirksamkeit der eingesetzten Instrumente eine notwendige Voraussetzung für organisationales Lernen dar (Carmeli und Schaubroeck 2008).

Erweitert und konkretisiert wurden diese Überlegungen insbesondere durch die Arbeiten zu organisationalem Lernen *während* und *zwischen* Krisen von Donald Moynihan (2008, 2009), auf die sich die folgende empirische Analyse stützt. Moynihan unterscheidet zwischen „kriseninternem Lernen“ und „krisenübergreifendem Lernen“. Kriseninternes Lernen („intracrisis learning“) beschreibt dabei die direkte Reflexion und Übernahme neuer Prozesse oder Anpassungen von Strukturen und Akteuren in der Auseinandersetzung mit der aktuellen Herausforderung. Krisenübergreifendes Lernen („intercrisis learning“) hingegen beschreibt das Ziehen von Lehren aus der Vergangenheit aus vergangenen bzw. überwundenen Krisen. Reflektiert wird insbesondere, ob traditionelle Schemata noch sinnvoll angewendet werden können, und welche organisationalen Anpassungen vor dem Hintergrund vergangener Krisenerfahrungen unternommen werden sollten (Hartley et al. 2013).

Es könnte also sein, dass eine Antwort auf die Frage, ob eine öffentliche Verwaltung ihre Leistungsfähigkeit in Krisenzeiten sicherstellen kann, davon abhängt, wann und wie welche Ausprägungen von Verwaltungsorganisation bzw. administrative Interaktionsform Anwendung finden (Schomaker und Bauer 2020; Nohrstedt et al. 2018). Die Erwartung solcher Netzwerk- und Lern-affinen Ansätze ist dementsprechend, dass öffentliche Verwaltungen Krisensituationen dann besser bewältigen, wenn sie, erstens, *innovative Lösungen produzieren* können; wenn sie, zweitens, den *Austausch über eine Interaktion in Netzwerken* forcieren; und wenn sie, drittens, *Kapazitäten vorhalten, um sich während und zwischen Krisen auf organisationales Lernen einzulassen*. Den Überlegungen von Donald Moynihan folgend können zwei zentrale Erwartungen abgeleitet werden, die die in der folgenden empirischen Analyse überprüft werden sollen. Eine erste Erwartung betrifft *kriseninternes Lernen („intracrisis learning“):* Verwaltungen, die während einer Krise ihre Netzwerke (im Sinne einer intensiven und guten Zusammenarbeit) mit Akteuren aus der Zivilgesellschaft, anderen Verwaltungseinheiten oder privaten Unternehmen erweitern und vertiefen und die durchgeführten Maßnahmen dokumentieren, weisen in Krisenzeiten eine höhere Leistung der Verwaltung auf. Eine zweite Erwartung fokussiert auf *krisenübergreifendes Lernen („intercrisis learning“):* Demnach sollten gerade solche Verwaltungen, die in Krisen auf bestehende Strukturen und Netzwerke zurückgreifen, also auf solche Strukturen, die bereits in vorhergehenden Krisen geschaffen wurden und weiter gepflegt wurden, eine höhere Verwaltungsleistung in Krisenzeiten aufweisen. Es ist die Überprüfung dieser beiden Zusammenhänge, die in der folgenden empirischen Untersuchung im Mittelpunkt stehen.

## Daten und Methode

Als Datenbasis der empirischen Analyse dienen zwei Online-Befragungen, die von den Autoren in Deutschland 2019 und 2020 durchgeführt wurden. Beide Befragungen fokussierten auf die Einschätzungen von Mitarbeitern der Kommunalverwaltungen und enthielten wortgleiche Fragebatterien zur Arbeitswelt, zum innerorganisatorischen Strukturwandel im Kontext der jeweiligen „Krise“, zu Vernetzungsaktivitäten mit anderen Behörden und externen Akteuren, zu Wissensspeicherung und -management sowie zu soziodemographischen Kennzahlen der jeweiligen Kommunen.

### Datenerhebung

Der erste Datensatz enthält die Ergebnisse einer Befragung, die über die Hochphase der Fluchtmigration zwischen 2015 und 2017 durchgeführt wurde. Die Befragung richtete sich konkret an alle deutschen Integrationsbeauftragten sowie an die Ausländer- und Sozialbehörden im Asylbereich in allen deutschen Großstädten mit mehr als 100.000 Einwohnern und in den Gemeinden mit den Anfangsbuchstaben D, E, H, K, N, R und S in allen Bundesländern (es erfolgte eine Zufallsauswahl der Buchstaben); zusätzlich wurden alle Landräte und Oberbürgermeister Deutschlands in die Stichprobe einbezogen. Die Befragung wurde zwischen Juli und November 2019 durchgeführt. Insgesamt wurden 2998 Personen eingeladen, von denen 750 an der Umfrage teilnahmen, was einer Rücklaufquote von 25 % entspricht.

Die zweite Umfrage fokussierte die erste Welle der COVID-19-Pandemie in Deutschland, die spätestens mit Beginn des nationalen Lockdowns am 22. März 2020 die lokale Ebene erreichte. Kontaktiert wurden neben allen deutschen Gesundheitsämtern auch Landratsämter und Oberbürgermeister sowie alle Bürgermeisterämter, in Kommunen, die mit dem Anfangsbuchstaben M beginnen.^1^ Der breite Zugang in diesem zweiten Survey schien angemessen, da die Pandemie nicht nur die Gesundheitsämter vor ungekannte Herausforderungen stellte, etwa bei der Kontaktnachverfolgung, sondern vielgestaltige Reaktionen in ganz unterschiedlichen Kontexten provozierte, wie etwa die Ausweitung von Homeofficeverpflichtung oder Abstandsgebote, die wiederum das Funktionieren nahezu aller Kommunalbehörden betrafen, etwa mit Blick auf interne Abläufe, Kundenkontakt oder der Serviceerbringung. Somit konnte durch ein entsprechend breit gewähltes Sample gewährleistet werden, dass tatsächlich behördenübergreifend herausgearbeitet werden kann, was die Determinanten hoher Leistungsfähigkeit sind, unabhängig von der Frage, welche Leistung eine Verwaltung im Einzelnen erbringt. Zwischen April und Mai 2020 nahmen von 1700 Gemeinden 364 Verwaltungsmitarbeiter an dieser Umfrage teil, was einer Rücklaufquote von rund 21 % entspricht.^2^

### Modell

Für eine empirische Überprüfung der theoretisch als relevant diskutierten Faktoren wurden verschiedene Ansätze verfolgt. Schätzmodelle wurden getrennt auf die beiden vorliegenden Datensätze angewandt, im Detail Logit-Modelle mit binären bzw. ordinal skalierten abhängigen Variablen, da die Annahmen für die Anwendung von OLS-Modellen nicht erfüllt sind. Somit wird als Koeffizient Exp (b) verwendet, der als „odd ratio“ (Effektgröße) verstanden werden kann. Diese gibt den Faktor an, um den sich das Wahrscheinlichkeitsverhältnis für das Eintreten eines Ereignisses gegenüber dem Nicht-Eintreten – also der Wahrscheinlichkeitsquotient – ändert, wenn die jeweilige unabhängige Variable ceteris paribus um eine Einheit erhöht wird; Exp (b) > 1 zeigt entsprechend ein erhöhtes Wahrscheinlichkeitsverhältnis für den Eintritt an, Exp (b) < 1 ein geringeres Wahrscheinlichkeitsverhältnis. Zudem wurden zur Robustheitsprüfung auch Probit-Modelle für alle Spezifikationen getestet. Darüber hinaus wurden verschiedene Tests auf Gruppenunterschiede durchgeführt (Mann-Whitney-U-Test, T‑Test).

#### Abhängige Variable

Zur Spezifizierung der „Leistung der öffentlichen Verwaltung“ bei der Bewältigung der Herausforderungen, die sich der spezifischen Verwaltung durch die jeweiligen Krisen stellen, wurden verschiedene Maße angewandt; alle Informationen für die Konstruktion der Variablen stammen aus den beschriebenen Datensätzen.^3^ Unter Verwendung einer Likert-Skala von 1‑5, die von „sehr gut“ (5) bis „sehr wenig“ (1) reichte, wurden den Befragten die folgenden Fragen gestellt.

- „Wie sehr stellen die Aufgaben zu Migration und Flucht (im zweiten Survey: die Aufgaben, die im Zuge der COVID-19-Pandemie anfallen), Ihrer Einschätzung nach, die Leistungs- und Innovationsfähigkeit der Kommunalverwaltung unter Beweis?“
- „Wie effektiv bewältigte Ihre Behörde aus Ihrer Sicht die Herausforderungen der hohen Zahlen an Geflüchteten (im zweiten Survey: die mit der aktuellen Pandemie verbundenen Herausforderungen)?“

Aus den Antworten auf die Fragen a) und b) wurden jeweils pro Datensatz die abhängigen Variablen kreiert. Im Detail wurde aus den ordinalskalierten Antworten auf die Fragen ein Index konstruiert, der ein niedriges bis hohes Leistungsniveau anzeigt. Aus diesem wurden verschiedene Spezifikationen von Dummyvariablen^4^ erstellt, welche den Wert 1 für eine hohe Leistungsfähigkeit der Verwaltung oder den Wert 0 für eine niedrige Leistungsfähigkeit annehmen.

Der Dummy „Verwaltungserfolg“ wird genutzt, sobald der Index einen Wert höher oder gleich 8 annimmt, also zumindest eine der Fragen mit einer hohen Einschätzung beantwortet wurde. Der Dummy „Verwaltungserfolg alternativ“ bildet ein höheres Leistungsniveau ab, da er ausschließlich für Indexwerte höher oder gleich 9 eintritt, somit wenn zumindest eine der Fragen mit „sehr hoch“, die andere mit „hoch“ beantwortet wurde. Zudem genutzt wurde für Kontrollzwecke ein Dummy für „Effektivität“, welcher nur Antworten auf Frage b) berücksichtigt und der den Wert 1 annimmt, sobald diese Frage mit „sehr effektiv“ oder „effektiv“ beantwortet wurde.^5^

#### Unabhängige Variablen

Zur Konstruktion der unabhängigen Variablen wurden für beide Datensätze aus den Antwortmöglichkeiten (Likert-Skala) der Befragungen Dummies erstellt, welche die Maßnahmen abbilden, die die öffentlichen Verwaltungen im Zusammenhang mit der jeweiligen Krise ergriffen haben. Die unabhängigen Variablen wurden jeweils für beide Datensätze gleich konstruiert, so dass eine komparative Analyse der Ergebnisse möglich ist.

##### Wissensmanagement:

Dummy „Dokumentation“: Wenn Erfahrungen aufgearbeitet, systematisch abgelegt oder in einer anderen Form weitergegeben wurden, damit Mitarbeitende der gleichen Behörde in künftigen Ausnahmesituationen darauf zurückgreifen können, nimmt die Variable den Wert 1 an, findet keine Dokumentation statt, den Wert 0.

##### Kooperation und Netzwerke:

Es wurden Dummies für die folgenden Indikatoren erstellt: „Qualität der Netzwerke zur Zivilgesellschaft“ sowie „Qualität der Netzwerke zu Verwaltungen“. Der jeweilige Dummy nimmt den Wert 1 an, sobald die Frage „Die Interaktion mit dem jeweiligen Akteur hat gut funktioniert“ mit „stimme voll und ganz zu“ oder mit „stimme zu“ beantwortet wurde; andernfalls 0. Darüber hinaus wurden Dummies für „Intensivierung der Netzwerke mit der Zivilgesellschaft“ und „Intensivierung der Netzwerke mit der Verwaltung“ erstellt; hierbei wird der Wert 1, sobald das Netzwerk in der jeweiligen Krise intensiviert wurde, ansonsten 0.

##### Vorbereitung („Preparedness“):

Für die folgenden Indikatoren wurden Dummies erstellt: „bestehende Kooperationen zu anderen Verwaltungen“ und „bestehende Kooperationen zu der Zivilgesellschaft“ sowie „bestehende Kooperationen zur Privatwirtschaft“. Die jeweiligen Dummies nehmen den Wert 1 an, sobald der Befragte angibt, dass seine Verwaltung bereits vor der „Flüchtlingskrise“ (respektive vor der Pandemie, in der „Flüchtlingskrise“) geschaffene Netzwerke mit dem jeweiligen Akteur nutzt und wiederbelebt hat, ansonsten 0. Darüber hinaus wurde für das COVID-19 Sample ein Dummy „Preparedness“ erstellt, der den Wert 1 annimmt, sobald zwei oder mehr der früheren Dummys den Wert 1 zeigen, und ansonsten 0.

## Ergebnisse

### Deskriptive Ergebnisse

**Figure 1:**
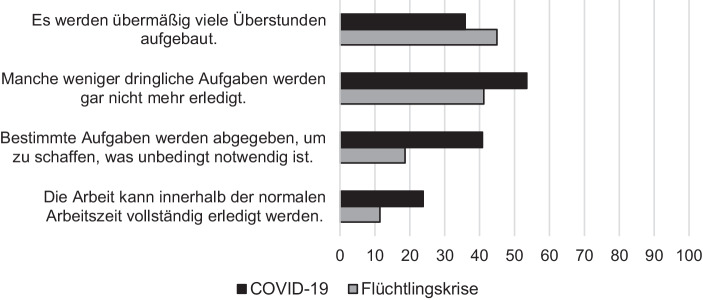

Zwar zeigte sich in beiden Umfragen, dass die jeweiligen Verwaltungen durchaus partiell aus- und auch überlastet waren (siehe Abb. 1). Bezogen auf die COVID-19-Pandemie trifft dies besonders auf die Gesundheitsämter zu.

**Figure 2:**
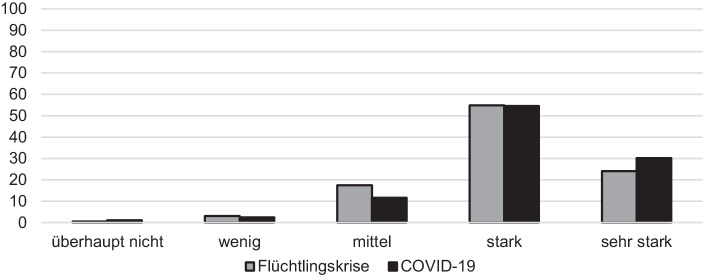

Dennoch gab ein großer Teil der Befragten an, dass die Aufgaben trotz der Krisensituation gut bewältigt werden konnten und dass die Krise die Leistungs- und Innovationsfähigkeit der Verwaltung unter Beweis stellt. Dies gilt sowohl im Kontext der Fluchtmigration als auch im Zuge der „ersten Welle“ der Pandemiebewältigung im Frühjahr 2020 (siehe Abb. 2).

**Figure 3:**
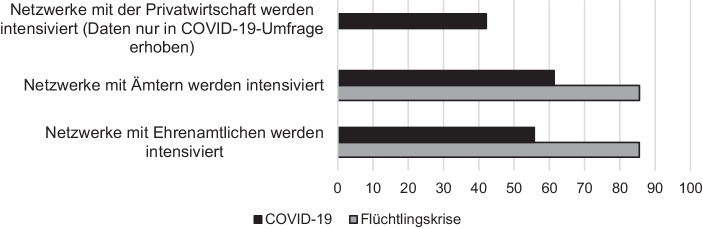

Dass Netzwerke eine entscheidende Rolle bei der Krisenbewältigung spielen, durfte erwartet werden (Geser 1997). Eine stärkere Vernetzung innerhalb einer Verwaltung sowie mit externen Akteuren wie anderen Ämtern oder ehrenamtlichen Helfern kann, wie referiert, dazu beitragen, Informationsverluste zu vermeiden und auf die Kapazitäten anderer zurückzugreifen (Löffler et al. 2015; Alford 2011; Bovaird 2007). Auch unsere Daten unterstreichen die Bedeutung von Netzwerken (siehe Abb. 3). In mehr als 80 % der Fälle wurde in der Hochphase der Fluchtmigration die Abstimmung mit anderen Ämtern und die Netzwerkarbeit mit Ehrenamtlichen qualitativ und/oder quantitativ ausgebaut. Auch während der COVID-19-Pandemie wurden in mehr als der Hälfte der Fälle die Netzwerke zu anderen Behörden und zu Ehrenamtlichen intensiviert.

**Figure 4:**
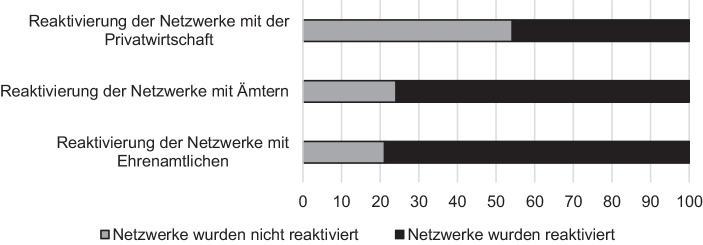

Ein Großteil dieser Netzwerke konnte aus der Flüchtlingskrise reaktiviert werden: In 79 % der Fälle für Netzwerke mit Ehrenamtlichen und 76 % der Fälle bzgl. Netzwerke zu anderen Ämtern konnte auf die verstärkten Netzwerkstrukturen seit der Hochphase der Fluchtmigration zurückgegriffen werden (siehe Abb. 4). Dieser Rückgriff auf bestehende Strukturen ohne langfristige Vorbereitungsaktivitäten und hohe Transaktionskosten ist von besonderer Relevanz im Krisenkontext, welcher oftmals von der Notwendigkeit zu schnellem Handeln gekennzeichnet ist.

**Figure 5:**
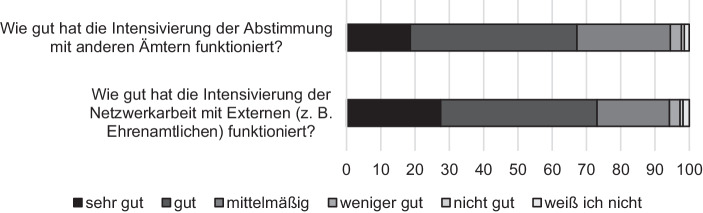

Die Netzwerkarbeit wurde vom Großteil der Befragten in beiden Krisensituationen als gut funktionierend bewertet, wobei in der Flüchtlingskrise die Qualität des Netzwerkausbaus als etwas besser wahrgenommen wurde (siehe Abb. 5 und 6).

**Figure 6:**
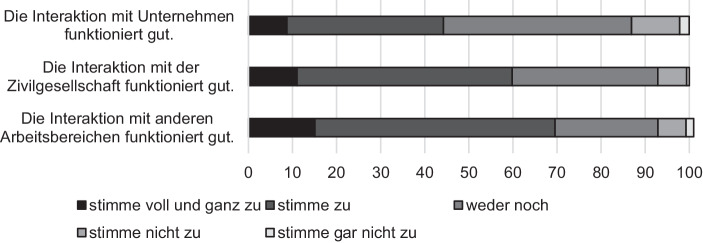

**Figure 7:**
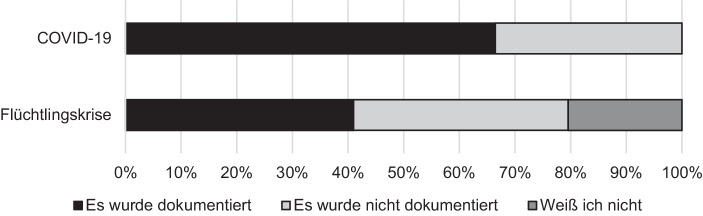

Zudem wurde erfasst, ob Erfahrungen aus der Krisensituation aufgearbeitet wurden, systematisch abgelegt oder in einer anderen Form weitergegeben wurden, damit in künftigen Ausnahmesituationen darauf zurückgegriffen werden kann (siehe Abb. 7). Diese Art der Dokumentation ist, wie oben ausgeführt, ein Teil des systematischen Wissensmanagements – dieses erhält Sachinformationen und Erfahrungswerte in der öffentlichen Verwaltung, selbst wenn beteiligte Mitarbeiter die Organisation verlassen (Probst et al. 2012; Sauter und Scholz 2015; Sutter 2016). Zudem können Kosten eingespart werden, wenn auf Erfahrungen zurückgegriffen werden kann und Doppelarbeit vermieden wird (Müller 2004).

Wissensmanagement ist in der deutschen öffentlichen Verwaltung, besonders im Vergleich zur Privatwirtschaft, wenig verbreitet (Bumiller et al. 2015; Fischer 2018). Dies legen auch unsere Daten nahe. Während der Hochphase der Fluchtmigration und danach wurde lediglich bei 41 % der Befragten Erfahrungen aufgearbeitet und weitergegeben; davon gaben 20 % an, dass die Aufarbeitung erst im Nachhinein durchgeführt wurde. Die Einschätzung des Defizits verschärft sich bei der Analyse der Kommentare aus den Umfragen. Diese lassen darauf schließen, dass es auch bei den durchgeführten Dokumentationen Probleme gibt, dort wird die „schlechte Dokumentation“ sowie die „Ungeordnetheit [der] digitalen Ablage“ beklagt.^6^ Etwas besser stellt sich die aktuelle Situation während der aktuellen COVID-19-Pandemie dar. Dokumentationen werden immerhin von zwei Dritteln (66,5 %) der Befragten aufgearbeitet, systematisch abgelegt oder weitergegeben; signifikante Unterschiede zwischen Gesundheitsämtern und den restlichen Befragten existieren nicht. Somit kann auf diese Erfahrungen in zukünftigen Ausnahmesituationen zurückgegriffen werden; dies ist ein wichtiges Signal in Richtung von Auf- und Ausbau von kommunalem Wissensmanagement.

### Determinanten administrativer Leistungsfähigkeit

Vor dem Hintergrund der diskutierten Überlegungen zu Verwaltungshandeln in Krisensituationen stellt sich die Frage, welche Faktoren zu einer hohen Leistungsfähigkeit beitragen. „Leistungsfähigkeit“ als abhängige Variable im Kontext verschiedener Krisen und Verwaltungen mag im Detail eine unterschiedliche inhaltliche Ausprägung annehmen – so kann etwa im Kontext von Migration die flächendeckende und qualitativ gute Betreuung von Geflüchteten gemeint sein, während es sich für ein Gesundheitsamt auf geglückte Kontaktnachverfolgung bezieht. Da aus theoretischer Sicht die Determinanten hoher Leistungsfähigkeit, nämlich Netzwerkpflege, -ausbau sowie systematische Wissensweitergabe, in beiden Surveys die gleichen sind, erscheint auch eine grundsätzliche Aussage über die Erfolgsfaktoren von Verwaltungsperformanz in Krisensituation über die beiden Samples möglich.

Grundsätzlich zeigen Tests auf Gruppenunterschiede, dass Verwaltungen, die auf bestehende Netzwerk zurückgreifen konnten und somit auf eine Krisensituation vorbereitet waren sowie eine hohe Qualität in ihrer Netzwerkzusammenarbeit mit anderen Verwaltungen und der Zivilgesellschaft aufweisen, im Durchschnitt in der Krise signifikant eine bessere Leistungsfähigkeit berichten, gemessen anhand der verschiedenen Spezifikationen für Verwaltungsleistung. Gleichzeitig zeigt sich kein signifikanter Unterschied zwischen Gesundheitsämtern und anderen Teilen der Verwaltung.

Die Ergebnisse der Schätzmodelle zeigen für beide Krisensituationen einen deutlichen Einfluss verschiedener unabhängiger Faktoren auf die Leistungsfähigkeit der öffentlichen Verwaltung in der Krise, gemessenen in den verschiedenen Spezifikationen – die Wahrscheinlichkeit für Effizienz und Leistungsfähigkeit von Verwaltungen steigt, sobald die jeweiligen Bedingungen erfüllt sind.

Indikatoren für Dokumentation sowie für die Nachhaltigkeit der Netzwerkarbeit – also die Reaktivierung bestehender Netzwerke und die Qualität der Zusammenarbeit und Vernetzung mit verschiedenen Akteuren – weisen signifikante positive Koeffizienten auf, die auf einen positiven Einfluss dieser Faktoren auf die Leistung hindeuten. Dies gilt für die Hochphase der Fluchtmigration (siehe Tab. 1) ebenso wie für die COVID-19-Pandemie (Tab. 2).

**Table 1:** 

	I	Ii	Iii	Iv	V
Variable	Verwaltungserfolg		Effektivität	Verwaltungserfolg alternativ	
Intercept	1,617 (0,0893)***	1,207 (0,1070)*	1,192 (0,2608)	0,055 (0,6062)***	0,382 (0,2318)***
Koordination Amt ex ante	1,806 (0,2413)**	1,621 (0,2466)**	–	–	0,594 (0,3869)
Koordination Ehrenamt ex ante	1,171 (0,297)	1,064 (0,2654)	–	–	1,835 (0,3773)*
Dokumentation	–	2,338 (0,1736)***	1,821 (0,2608)***	1,630 (0,2985)*	1,920 (0,2862)**
Qualität der Netzwerke zur Zivilgesellschaft	**–**	–	1,786 (0,2768)**	2,355 (0,4674)*	–
Qualität der Netzwerke zu Verwaltungen	**–**	–	1,951 (0,2610)***	4,977 (0,4377)***	–
*N*	*682*	*678*	*409*	*227*	*227*

Signifikanzlevel *** 1 %, ** 5 %, * 10 %

Standardfehler in Klammern. Dargestellt als Koeffizient wird Exp (b)

**Table 2:** 

	i	Ii	Iii	Iv	v
Variable	Verwaltungserfolg	Effektivität	Verwaltungserfolg alternativ		
Intercept	1,571 (0,4835)	0,824 (0,9018)	0,108 (0,3702)***	0,379 (0,2318)***	0,162 (0,3370)***
Qualität der Netzwerke zu Verwaltungen	25,773 (0,8638)***	13,476 (0,8091)***	3,566 (0,3415)***	–	3,701 (0,3425)***
Qualität der Netzwerke zur Zivilgesellschaft	–	1,121 (0,4800)	1,691 (0,2746)*	–	1,839 (0,2820)**
Preparedness gesamt	–	2,688 (0,5097)**	–	–	1,621 (0,2613)*
Intensivierung der Netzwerke zur Zivilgesellschaft	–	–	–	1,700 (0,2677)**	–
Intensivierung der Netzwerke zur Privatwirtschaft	–	–	–	0,847 (0,2694)	–
Intensivierung der Netzwerke zu Verwaltungen	**–**	**–**	**–**	1,719 (0,2631)**	**–**
Dokumentation	**–**	–	2,168 (0,2834)***	**–**	**–**
*N*	*101*	*101*	*289*	*289*	*289*

Signifikanzlevel *** 1 %, ** 5 %, * 10 %

Standardfehler in Klammern. Dargestellt wird als Koeffizient Exp (b)

Im Sample zur COVID-19-Pandemie weisen zudem die Indikatoren für einen Ausbau und die Intensivierung von Netzwerken und Kooperationen mit der Zivilgesellschaft und anderen Ämtern signifikante Koeffizienten auf, die auf einen positiven Einfluss dieser Faktoren auf die Leistung hindeuten (siehe Tab. 2; dies gilt nicht für einen Ausbau der Kooperation mit Unternehmen, der entsprechende Indikator bleibt insignifikant).

Es ergibt sich somit mit Blick auf die empirischen Ergebnisse folgendes Bild: Netzwerke sind hilfreich bei der Bewältigung der Krise, dies gilt sowohl für den Ausbau neuer Kooperation wie auch für die Reaktivierung bzw. Intensivierung bestehender Kooperationen. Darüber hinaus ist jedoch die *Qualität* dieser Zusammenarbeit von besonderer Bedeutung – funktioniert die Kooperation gut, so steigt die Wahrscheinlichkeit für Leistungsfähigkeit und Effizienz der Verwaltung. Wissensmanagement im Sinne der Dokumentation von Prozessen und Ergebnissen ist zudem ein relevanter Faktor für eine effiziente Bewältigung von Krisensituationen im Sinne einer hohen Leistungs- und Innovationsfähigkeit der Verwaltung.

Werden die Ergebnisse vor dem Hintergrund der theoretischen Überlegungen interpretiert, so lässt sich schlussfolgern, dass sowohl das *Lernen innerhalb von Krisen* als auch das *Lernen zwischen Krisen* relevant ist, um auch in Ausnahmesituationen eine hohe Leistung der öffentlichen Verwaltung zu erreichen oder aufrechtzuerhalten, und dass sich beide Konzepte bis zu einem gewissen Grad ergänzen. Dies scheint sowohl für spezielle Verwaltungen – im Falle der aktuellen COVID-19-Pandemie für die Gesundheitsämter – als auch für Behörden allgemein zu gelten; die Ergebnisse lassen jedenfalls nicht auf signifikante Unterschiede schließen.

Es kann festgehalten werden, dass *kriseninternes Lernen* im Sinne der Dokumentation hilfreicher Praktiken für den Einsatz innerhalb einer Krise sowie die Zusammenarbeit und Vernetzung mit Interessengruppen eine hohe Leistungsfähigkeit zu sichern vermag. Der Ausbau von Netzwerken und insbesondere die Qualität der Zusammenarbeit sind an dieser Stelle von Bedeutung. In beiden Krisen hat eine gute und intensive Zusammenarbeit mit anderen Verwaltungen und Akteuren aus der Zivilgesellschaft innerhalb der Krise einen bedeutenden positiven Effekt auf die Gesamtleistung der Behörden. Zudem sind Spannungen zwischen Effizienz und Vernetzung nicht zu erkennen, sondern das Gegenteil scheint der Fall zu sein. Die Nutzung und der Rückgriff auf funktionierende Netzwerke übertreffen hierarchische Lösungen, und die Stabilität und Qualität von Netzwerken ermöglichen mehr Effektivität (Provan und Kenis 2008; Moynihan 2009). Daneben ist es *krisenübergreifendes Lernen*, welches eine Vorbereitung auf neue Krisensituationen erleichtert – dies entsteht etwa durch die Reaktivierung erfolgreicher Kooperationen und Netzwerke, aber auch durch den Rückgriff auf dokumentiertes Wissen (Moynihan 2008; Kettl 2006).

## Fazit

Der vorliegende Beitrag analysierte im Rückgriff auf Perzeptionsdaten von Mitarbeitern deutscher Kommunalverwaltungen die Bewältigung von Krisen als Ausnahmesituationen mit Überforderungspotenzial für die betroffenen Behörden (Boin und Lodge 2016). Hierzu wurden Surveys über den Umgang mit der Fluchtmigration nach Deutschland in den Jahren 2015 bis 2017 sowie mit der ersten Welle der COVID-19-Pandemie im Frühjahr 2020 durchgeführt. Während die konkrete Bedeutung von „hoher Leistungsfähigkeit“ im Detail von der jeweiligen Funktion der Verwaltung abhängt (und für ein lokales Gesundheitsamt bei der COVID-19-Pandemie natürlich eine andere Bedeutung hat als für eine Ausländerbehörde in der Hochphase der Fluchtmigration), vermag die vorliegende Studie dennoch Bestimmungsfaktoren für hohe administrative Leistungsfähigkeit auf einer allgemeineren Ebene zu beleuchten – und damit vergleichbar zu machen. Demnach gilt, dass dort, wo deutsche Kommunalverwaltungen in diesen Ausnahmesituationen gut zurechtkamen, immer auch funktionsfähige Netzwerkstrukturen, ausgiebige Dokumentationsverfahren sowie die Fähigkeit, auf frühere Erfahrungen zurückzugreifen, beobachtet werden konnten. Es waren also Netzwerk‑, Dokumentations- und Erinnerungs-Strategien, die den Verwaltungen halfen, ihre krisenspezifischen Aufgaben zu meistern. Damit stützt unsere Analyse die These, dass die Wahrscheinlichkeit einer erfolgreichen administrativen Krisenreaktion mit der Fähigkeit der Verwaltung steigt, frühere Erfahrungen zu nutzen und bezogen auf das konkrete Akteursumfeld inklusiv zu agieren. Intrakrisen- und Interkrisen-Lernen sind demnach eng miteinander verbunden, wobei ersteres als eine Voraussetzung für letzteres gelten kann (Punz 2020).

Weitere Forschung zur Rolle von Intrakrisen- und Interkrisen-Lernen erscheint daher angezeigt – besonders im Hinblick auf Wirkungszusammenhänge in verschiedenen sozialen und politisch-administrativen Kontexten sowie zum Zusammenhang zwischen den beiden Lernarten (so auch Broekema 2016). Zunächst sollte aber das Potenzial eines reflexiven und adaptiven Einsatzes von Multi-Akteurs-Netzwerken zur Bewältigung von außergewöhnlichen Situationen systematisch untersucht werden. Ferner, da eine erfolgreiche Leistung in Krisensituationen anscheinend vom Wissensaustausch und von der Fähigkeit abhängt, den Nexus öffentlich-privater Akteure zu koordinieren, sollte die Untersuchung der *Bedingungen von Kommunikation* und der *Koordination über Domänengrenzen hinweg* für Verwaltungswissenschaftler zentral bleiben.

Die zweite und dritte Welle der COVID-19-Pandemie, die außerhalb des Untersuchungsrahmens dieser Studie bleiben mussten, unterstreichen einerseits bedauerlicherweise die praktische Relevanz der Erforschung von Verwaltungslernen in Krisensituationen, andererseits bieten sie aber auch weitere Gelegenheiten zu vergleichenden empirischen Untersuchungsdesigns. Mit dem Ziel unsere öffentliche Verwaltung resilienter zu machen, sollte sich die politikwissenschaftliche Verwaltungswissenschaft dieser Agenda unbedingt annehmen.

## Footnotes

1 Bei der Umfrage zum kommunalen Flüchtlingsmanagement korrespondieren die 750 Teilnahmen mit der Größe der Bundesländer. Ausreißer stellen lediglich Berlin mit keinem, Bremen mit einem und das Saarland mit zwölf Teilnehmern dar. 16 % arbeiten in Kleinstädten, 24 % in Großstädten und ein Drittel in Landkreisen. An der Studie zur COVID-19-Pandemie nahmen Teilnehmer aus allen Bundesländern mit Ausnahme von Sachsen-Anhalt teil. Auch bei dieser Umfrage arbeiten 16 % der Befragten in Kleinstädten, 15 % in Großstädten und die Hälfte der 364 Befragten in Landkreisen; insgesamt nahmen 81 Personen aus den deutschen Gesundheitsämtern teil. Rund die Hälfte der Befragten (59 %) haben leitende Positionen. Aufgrund des kleinen N dürfen die Ergebnisse nicht überinterpretiert werden – dennoch haben diese Daten Aussagekraft, da kaum Studien zur Lage in den deutschen Gesundheitsämtern vorliegen (Ausnahmen etwa Grill und Leurs 2020).

2 Abhängig von den konkreten Antworten auf „Filterfragen“ – z. B. ob bestimmte Netzwerke in der jeweiligen Krise neu belebt wurden – konnten nicht alle Fälle aus der jeweiligen Umfrage in die Schätzmodelle einbezogen werden. Diese Tatsache erklärt Abweichung in der Anzahl von Fällen, die in den Schätzungsmodellen berücksichtigt wurden, verglichen mit der Gesamtstichprobengröße. Darüber hinaus ist die Auswahllogik – weniger Verwaltungen, Konzentration auf Gesundheitsämter, die mitten in einer Gesundheitskrise angesprochen werden – für die insgesamt geringere Fallzahl in der zweiten Umfrage verantwortlich.

3 Generell ist anzumerken, dass die Variablen in ihren verschiedenen Spezifikationen auf selbstberichteten Wahrnehmungsdaten basieren und die Antworten die eigene Einschätzung der Leistungsfähigkeit der Verwaltung des Befragten in der Krise darstellen. Die Vor- und Nachteile der Wahl dieses empirischen Zugangs könnten an dieser Stelle ausführlich diskutiert werden. Siehe hierzu grundsätzlich Brewer (2006). Verzerrungen in Richtung sozialer Erwünschtheit oder Überschätzung der Ergebnisse aufgrund fehlender neutraler Informationen sind bei organisatorischen Leistungen, die von Personen aus der Organisation relativ genau beurteilt werden können, weniger wahrscheinlich, als wenn die persönliche Leistung im Mittelpunkt stünde. Zur Verwaltungsleistung ist die Erhebung von Perzeptionsdaten gängige Praxis (beispielsweise Worrall et al. 2000; Pandey et al. 2007). Dennoch wäre die Erhebung von Strukturdaten – wenigstens als Ergänzung – sicherlich wünschenswert. Die Ausgestaltung der behördlichen Aufgaben und Reaktionsweisen lässt es jedoch wenig wahrscheinlich erscheinen, durch ein anderes Vorgehen kurzfristig zu vergleichbaren Daten zu kommen. Der Fokus auf Perzeptionsdaten erscheint daher – zu diesem Zeitpunkt – der einzig gangbare Weg. Dennoch kann man von einer internen Validität ausgehen und gültige kausale Schlussfolgerungen ziehen – da erstens die „Ursache“ der „Wirkung“ zeitlich vorausgeht (zeitliche Präzedenz), zweitens die „Ursache“ und die „Wirkung“ tendenziell zusammen auftreten (Kovariation) und drittens, da es keine wissenschaftlich begründbaren alternativen Erklärungen für die beobachtete Kovariation gibt (Nicht-Verfälschung). Externe Validität kann als gegeben angesehen werden, wenn die Anwendung der Schlussfolgerungen einer wissenschaftlichen Studie außerhalb des Kontexts dieser spezifischen Studie möglich ist.

4 Eine Dummyvariable bezeichnet eine Variable, die nur die Ausprägungen 0 und 1 besitzen kann – entweder liegt eine Ausprägung vor, dann wird diese Variable zu 1, oder sie liegt nicht vor, dann nimmt die Variable den Wert 0 an.

5 Die Umwandlung in eine Dummyvariable wird vorgenommen, um binäre Modelle anwenden zu können. Im Vergleich verschiedener Modelle wiesen diese die größte statistische Passung auf, so dass die entsprechenden Modelle für die Darstellung ausgewählt wurden. Es wurden für eine Prüfung der Robustheit der Ergebnisse auch sog. „Kleinste-Quadrate Modelle“ (OLS) geschätzt, aufgrund der Ordinalskalierung der Daten und der fehlenden Normalverteilung haben diese jedoch eine geringe Passung, so dass die Ergebnisse nicht dargestellt werden.

6 Dokumentiert wurden, nach den Erkenntnissen der Autoren, Erfahrungen der unmittelbaren Krisenreaktion der Verwaltungen. Was genau dokumentiert wurde, konnte aufgrund der Komplexität des Krisenhandelns der öffentlichen Verwaltungen nicht abgefragt werden.
